# Physical collections, virtual classes: creating digital access to anatomy models for remote learning

**DOI:** 10.5195/jmla.2021.1354

**Published:** 2021-10-01

**Authors:** Daniel McCallum, Laura Burt-Nicholas

**Affiliations:** 1 mccallumd384@cod.edu, Media Lab Supervisor, College of DuPage Library, Glen Ellyn, IL; 2 burt-nicholasl@cod.edu, Reference Librarian/Associate Professor, Biological and Physical Sciences Liaison, College of DuPage Library, Glen Ellyn, IL

The College of DuPage library, serving a community college in the western suburbs of Chicago, embarked upon a project to make its nearly 300-item anatomical model collection accessible to anatomy and physiology students over summer 2020. This project began when the college courses and student services went virtual due to concerns about the COVID-19 pandemic. In the absence of access to the physical models collection, library media lab staff created images and recorded videos of anatomy and physiology faculty as they taught important model features.

The library media lab supervisor was instrumental in designing a plan to capture photos and video with studio equipment from the media lab and to host the content on a third-party media platform that restricted access to the college community. This allowed faculty to focus on content expertise, while technical processes were handled by library media lab staff in consultation with college IT and instructional design experts.

All equipment used for this project is available for use by students in the library's media lab. The only equipment purchased for this project was a black backdrop and an overhead camera mount. Videos and photos were primarily taken from a top-down perspective, as most models were easier to display lying flat. Video was captured with a Canon C100 Mk II camera, and the video signal was output to an Atomos Ninja Flame monitor. The monitor allowed faculty to see the positioning of models in the camera's frame, and it recorded the video signal directly to solid state drives (SSD), which made it easier to back up media files. All video was recorded at a resolution of 1080p and at a frame rate of 23.98.

Photos were captured with a Canon EOS M50. All photos were captured as RAW files on SD cards.

During recording or shooting, all equipment was plugged into AC outlets to avoid changing batteries.

**Figure 1:**
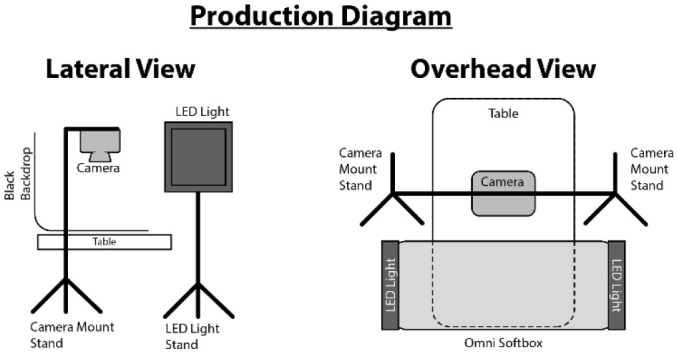
Production diagram

The cameras were mounted on a Glide Gear OH 75 overhead pole rig for top-down shots. The only lighting sources used were two Dracast LED 1000 lights that were both connected to an Aputure Omni Softbox and hung horizontally across the table used for shooting. This provided a soft, ambient light that required little adjustment between models. (Figure 1).

Audio was recorded directly to the C100 with a Rode condenser microphone on a mic stand.

COVID-19 safety precautions were followed at all times, including wearing masks and maintaining social distance. Remote camera features were used to maintain social distance. The C100 was operated by laptop over WiFi, and the EOS M50 was operated via the Canon Camera Connect app on an Android device. Both cameras could be controlled in full manual mode this way, without having to remove them from the overhead rig.

Production took seven days and was carried out in an empty anatomy classroom. Equipment was operated by the library's media lab supervisor, and two full-time anatomy faculty presented and narrated the videos. The classroom was large enough to allow the media lab supervisor and both anatomy faculty to be present and maintain social distance guidelines.

The entire postproduction process was done remotely. Photos were shared through Microsoft OneDrive, and videos were uploaded to Yuja, a third-party video hosting platform. The anatomy faculty sorted photos and indicated which ones needed editing and which could be deleted.

Photos were edited in Adobe Lightroom, with detailed edits made in Photoshop. Edits consisted primarily of adjustments to contrast, exposure, and color balance. Some models required more extensive edits to remove distracting elements such as labels and magnets on the models and foam used to prop up models.

Video editing was done in Adobe Premiere Pro. This process included cutting together multiple takes, making adjustments to exposure and color balance, adding titles, and cropping out backgrounds visible behind the backdrop. The media lab supervisor edited videos and photos, and two full-time anatomy faculty provided input on revisions.

To increase accessibility, Yuja's auto-captioning service was used for all videos. Anatomy faculty reviewed videos and made corrections to caption files where errors were found in the auto-captions.

The college's Learning Technologies department assisted with creating photo slideshows and with designing a process to post photos and videos to the Blackboard LMS. Over four hundred students have accessed the collection since its creation. Further benefits were that the library faculty and media lab staff could collaborate to expand access to library collections and deliver timely content to students. Limitations included COVID-19 safety precautions, which limited the amount of time and number of people who could participate in the production process, and ultimately, the number of models that could be captured.

